# Design and applications of a clamp for Green Fluorescent Protein with picomolar affinity

**DOI:** 10.1038/s41598-017-15711-z

**Published:** 2017-11-24

**Authors:** Simon Hansen, Jakob C. Stüber, Patrick Ernst, Alexander Koch, Daniel Bojar, Alexander Batyuk, Andreas Plückthun

**Affiliations:** 1 Department of Biochemistry, University Zürich, Winterthurerstrasse 190, 8057 Zürich, Switzerland; 2 Present Address: Department of Early Discovery Biochemistry, Genentech, 1 DNA Way, South San Francisco, California, 94080 USA; 3 Present Address: Department of Biosystems Science and Engineering, ETH Zürich, Mattenstrasse 26, 4058 Basel, Switzerland; 4 Present Address: Linac Coherent Light Source, SLAC National Accelerator Laboratory, 2575 Sand Hill Road, Menlo Park, California 94025 USA

## Abstract

Green fluorescent protein (GFP) fusions are pervasively used to study structures and processes. Specific GFP-binders are thus of great utility for detection, immobilization or manipulation of GFP-fused molecules. We determined structures of two designed ankyrin repeat proteins (DARPins), complexed with GFP, which revealed different but overlapping epitopes. Here we show a structure-guided design strategy that, by truncation and computational reengineering, led to a stable construct where both can bind simultaneously: by linkage of the two binders, fusion constructs were obtained that “wrap around” GFP, have very high affinities of about 10–30 pM, and extremely slow off-rates. They can be natively produced in *E. coli* in very large amounts, and show excellent biophysical properties. Their very high stability and affinity, facile site-directed functionalization at introduced unique lysines or cysteines facilitate many applications. As examples, we present them as tight yet reversible immobilization reagents for surface plasmon resonance, as fluorescently labelled monomeric detection reagents in flow cytometry, as pull-down ligands to selectively enrich GFP fusion proteins from cell extracts, and as affinity column ligands for inexpensive large-scale protein purification. We have thus described a general design strategy to create a “clamp” from two different high-affinity repeat proteins, even if their epitopes overlap.

## Introduction

Since the first demonstration that the green fluorescent protein (GFP) derived from *Aequorea victoria* can be used to label proteins *in vivo* and to directly study structures and processes in cells^1^, it has become an indispensable tool in cell biological research (reviewed in refs^2,3^). Applications became even broader through the development of a series of monomeric fluorescent proteins (FPs) with different spectral properties^4–10^. Nowadays, thousands of functionally tested GFP-fusion constructs in cell lines or living organisms exist. To study such constructs, reagents with high specificity and affinity are of great interest. They can, for example, be used to detect or enrich GFP-fusions^11,12^, to relocalize or manipulate GFP-tagged proteins in living organisms^13,14^, or to deliver stable organic fluorophores for super-resolution microscopy^15^, and they may allow to bridge the gap between light and electron microscopy^16,17^.

Besides studying processes in living cells, GFP has also been used to characterize the functional overexpression of proteins, often with the final goal of investigating the structural properties of these proteins. Several approaches have been worked out to detect correctly folded proteins during expression by directly fusing GFP to a protein of interest^18^. More sophisticated approaches use Förster resonance energy transfer (FRET) between two FPs at both termini of the protein of interest^19^ or employ self-complementing split GFP variants^20^. These approaches have been expanded to membrane proteins which are often difficult to express^21–23^. GFP was also shown to be useful for detecting the oligomeric state of a protein in crude expression extracts using fluorescence-detection size exclusion chromatography^24^. Hence, an inexpensive affinity resin to purify proteins expressed as GFP-fusions would also be very helpful.

Several specific detection reagents for GFP have been developed. The first were based on antibodies, of which several poly- and monoclonal ones are commercially available, and later on single-domain binding proteins like camelid-antibody-derived variable heavy chain fragments (V_H_H, also called nanobodies)^11,25,26^, designed repeat proteins based on the natural HEAT-repeat protein family (αRep)^12,27^, and designed ankyrin repeat proteins (DARPins)^14^. DARPins are engineered proteins that consist of 4–5 structurally similar repeats that stack on top of each other, forming a compact protein domain. The repeats that contain the N- or C-terminus differ from the middle repeats; their exposed surfaces are more hydrophilic, protecting the hydrophobic protein core from solvent exposure. The terminal caps are named N- and C-cap, respectively. DARPins combine all potential advantages of single domain binders over antibodies: they are small in size (ca. 18 kDa), possess high biophysical stability, have very high expression yields in *E. coli*, can easily be genetically encoded for *in vivo* applications and are devoid of disulphides and hence well suited for applications where they are expressed in the reducing cytoplasm^28,29^. Moreover, their concave shape and rigidity facilitates the engineering of more advanced molecules as demonstrated here.

Here we describe the structure-guided design and characterization of “GFP-clamps” that are based on the two previously described GFP-specific DARPins 3G124 and 3G61^14^. Structural investigations showed that these DARPins bind to different but slightly overlapping epitopes^30–32^. In a first step, stable truncated versions of 3G61 were designed that, through truncation and surface engineering, were able to bind GFP simultaneously with 3G124. Linking of 3G124 with the truncated and reengineered 3G61 produced GFP-clamps with very high affinities. Biophysical characterization showed that the GFP-clamps were very stable, monomeric and easily produced in *E. coli*, allowing for many new applications, where these properties are important. We demonstrate a few of them here: GFP-clamps were used to immobilize target proteins on surface plasmon resonance (SPR) chips, an inexpensive GFP affinity column was produced which can be used to purify GFP-tagged proteins of interest even on a large scale; fluorescently labelled GFP-clamps were used as monovalent tight-binding detection reagents in flow cytometry; and in pull-down experiments GFP-fusions could be specifically enriched.

## Results

### Structure-guided design approach to remove epitope overlap of 3G61 and 3G124

**Figure 1:**
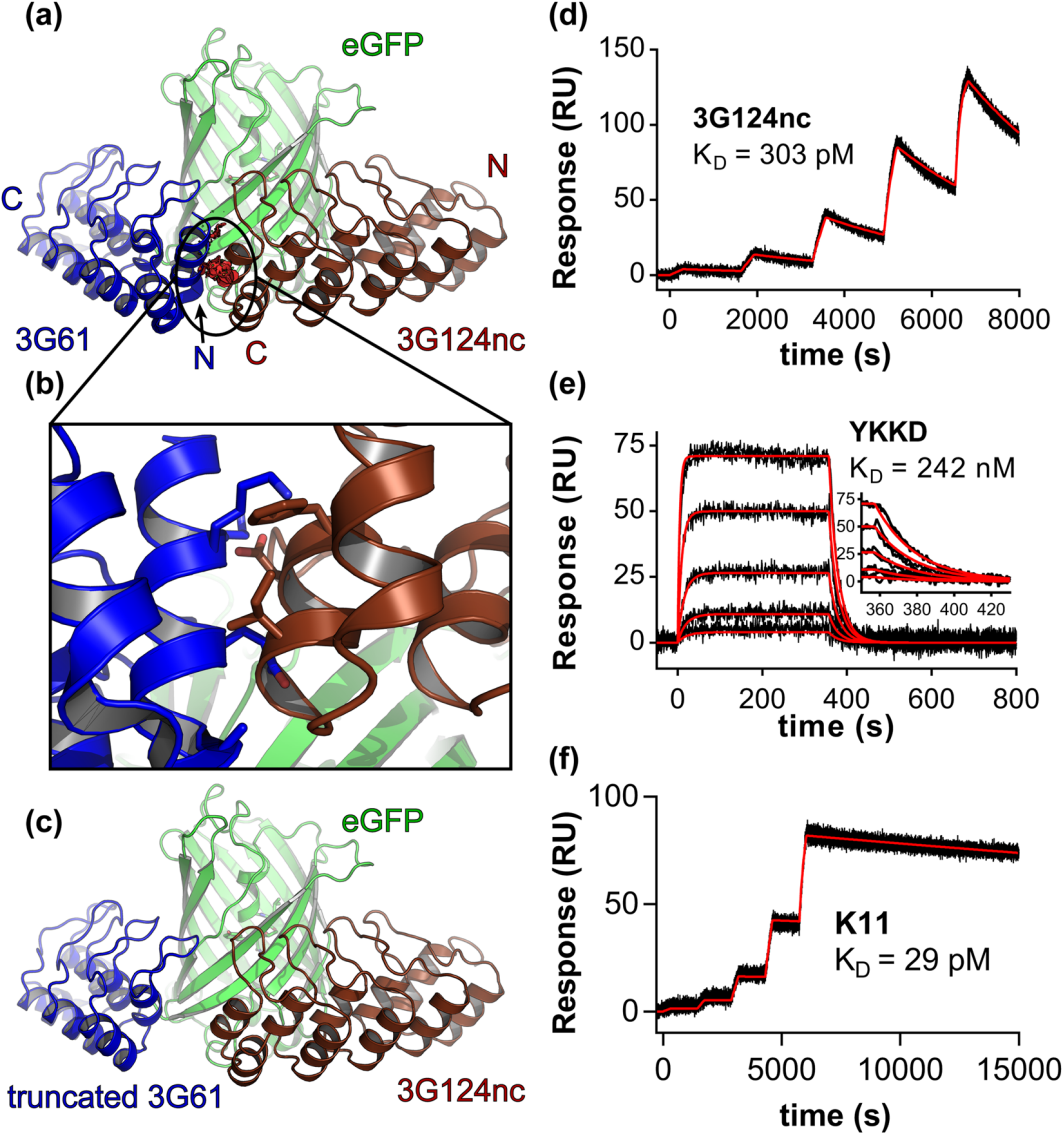
Design of GFP-clamps. (**a**) Superposition of 3G61 and 3G124nc, determined in individual complex structures, on GFP reveals clashes (red cylinders). (**b**) Close-up view of the clash, side chains that clash with main chain atoms are shown as sticks. (**c**) Clashes can be removed by truncation of the N-cap of 3G61. (**d**) Affinity determination of 3G124nc to GFP by SPR. (**e**) Affinity determination of YKKD, a version of truncated 3G61; the inset shows a close-up view of the dissociation phase. (**f**) Affinity determination of GFP-clamp gc_K11 which is a fusion of the two proteins above.

**Figure 2:**
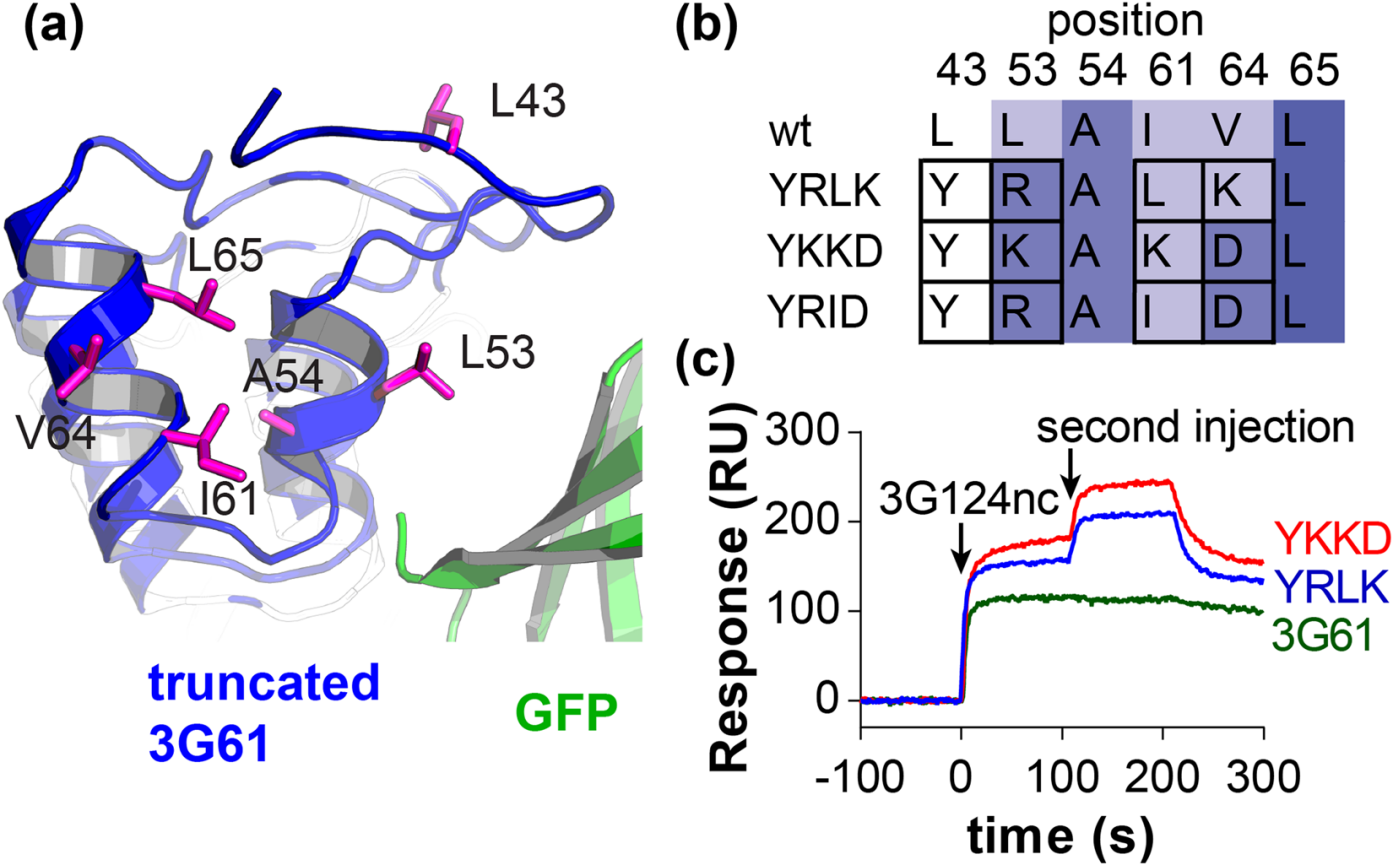
Computational redesign of truncated 3G61. (**a**) View of the newly exposed hydrophobic surface after N-cap truncation of 3G61. All residues allowed to be mutated in Rosetta simulations are shown as magenta sticks. (**b**) Mutations of selected residues before (wt) and after simulations. Rosetta energy scores are represented as a white-blue gradient where white represents neutral and blue good Rosetta scores. Black frames show residues that were mutated with respect to the wild type (wt) and used to name the truncated constructs. (**c**) SPR co-injections show that, in contrast to 3G61, YKKD and YRLK were able to bind GFP simultaneously with 3G124nc.

Structures of 3G61 and 3G124^14^ in complex with GFP were determined previously. 3G61 was crystallized as a rigid fusion to β-lactamase^30^ (PDB ID: 5AQB) whereas 3G124 was fused rigidly to a second DARPin^32^ (PDB IDs: 5LEL and 5LEM). Structures of the unfused DARPins 3G61 and 3G124nc (nc indicates the use of the optimized C-cap (Mut5)^33,34^) in complex with enhanced GFP (eGFP)^35,36^ have now been determined (PDB IDs: 5MA6, 5MA8 and 5MAK) and confirmed the epitopes (Fig. 1). The N-cap of 3G61, which was only partially visible in the structure of the β-lactamase fusion, was completely resolved in the unfused 3G61:eGFP structure. Superposition of the unfused structures revealed two different epitopes on GFP with an arrangement where the C-terminus of 3G124 is in very close proximity to the N-terminus of 3G61. However, clashes between the N-cap of 3G61 and the C-cap of 3G124 were detected in the structural alignment (Fig. 1(a) and (b)), which would preclude a direct linkage. This was corroborated by surface plasmon resonance (SPR) co-injection experiments where it was found that 3G61 and 3G124 are indeed not able to bind GFP simultaneously (Fig. 2(c)).

In silico truncation of the 3G61 N-cap removed all clashes of main chain atoms in the superposition; only minor clashes between side chains of Glu45 (3G61) and Phe145 (3G124nc) remained (Fig. 1(c)). We speculated that these clashes would disappear when the residues adopt different rotamers. Hence, we reasoned that it might be possible to make a fusion of the DARPins that wraps around half a GFP molecule. We chose the name “GFP-clamps” for such constructs that connect full-length 3G124nc to an N-cap-truncated 3G61 via flexible linkers.

### Design of truncated 3G61 using Rosetta

Truncation of the N-cap exposes a surface to the solvent that was previously buried in the hydrophobic core. For the stability of the truncated DARPin it was necessary to introduce mutations at this surface to regain a similar hydrophilicity as of the original N-cap, while retaining the native secondary structure. Truncated constructs were modelled with the fixed backbone design application of Rosetta version 3.4^37,38^. Six exposed hydrophobic residues, namely Leu43, Leu53, Ala54, Ile61, Val64 and Leu65 were allowed to mutate to any residue except Cys in a model of 3G61 with a truncated N-cap in complex with GFP during Rosetta simulations (Fig. 2(a)). Analysis of the sequences and energy scores of output structures showed that the energy scores were very similar for all output structures and sequences converged to three different solutions. Ala54 and Leu65 remained unchanged, whereas Leu43 was always mutated to Tyr43. Leu53, Ile61 and Val64 were mutated to Arg53, Leu61 and Lys64 (YRLK) or Lys53, Lys61 and Asp64 (YKKD) or Arg53, Ile61 and Asp64 (YRID), respectively (Fig. 2(b), SI Fig. 1).

### Characterization of truncated 3G61 and 3G124nc

**Figure 3:**
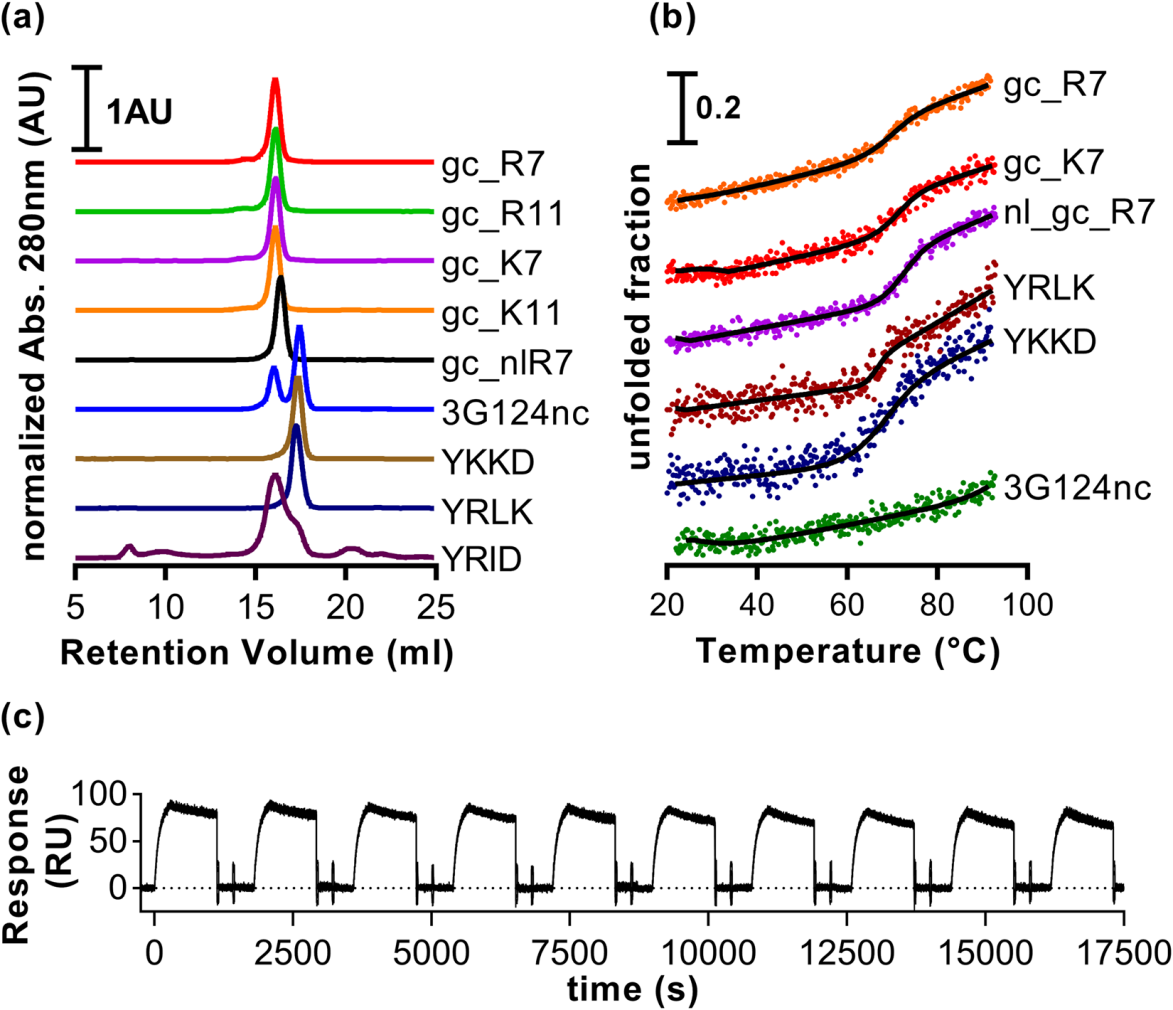
Characterization of individual DARPin domains and GFP-clamps. (**a**) SEC-MALS experiments. Only elution profiles are shown and the measured molecular weights are given in Table 1. (**b**) Thermal denaturation of several constructs monitored by circular dichroism at 222 nm (dots) with fits, extracted melting temperatures are given in Table 1. (**c**) Repeated injections of GFP and regeneration of a SPR chip functionalized with biotinylated GFP-clamps.

The variants YRLK, YKKD and YRID were cloned, expressed and purified from *E. coli* via immobilized metal ion affinity chromatography (IMAC). Their oligomeric state was determined by size exclusion chromatography monitored by UV and multi-angle static light scattering detectors (SEC-MALS). YRLK and YKKD showed symmetric single elution peaks and the determined molecular weight (MW) corresponded well with the theoretical molecular weight, whereas YRID eluted as several peaks. The main peak corresponded to a dimer, but additional peaks were also observed, the earliest appearing in the void volume. Storage at 4 °C for some days led to complete elution of YRID in the void volume (data not shown), hence YRID was regarded as aggregated and was excluded from further investigation (Fig. 3(a), Table 1).

**Table 1:** SEC-MALS and thermal denaturation.

Protein	Size exclusion chromatography - multiangle static light scattering					thermal denaturation
	Retention volume (ml)	theoretical MW^a^ (kDa)	measured MW^b^ (kDa)	ratio^c^	oligomeric state^d^	melting point
gc_R7	16.08	31.36	30.9	0.99	m	68.7 °C
gc_R11	16.1	31.61	30.2	0.96	m	n.d.
gc_K7	16.11	31.33	30.2	0.96	m	70.5 °C
gc_K11	16.09	31.59	30.3	0.96	m	n.d.
nl_gc_R7	16.38	31.71	30.2	0.95	m	71.8 °C
3G124nc	16.02/17.44^e^	16.95	31.7/15.9^e^	1.87/0.94^e^	d/m^e^	>92 °C
YKKD	17.34	14.27	14.4	1.01	m	66.6 °C
YRLK	17.25	14.30	15.7	1.10	m	66.0 °C
YRID	16.1^f^	14.28	27.5^f^	1.93	d/agg	n.d.

n.d.: not determined. ^a^Calculated from the sequence. ^b^Measured by MALS. ^c^ratio = measured MW/theoretical MW. ^d^m: monomeric; d/m: mixture of dimer and monomer; agg: aggregate. ^e^Data for the first and second peak, respectively. ^f^Data for the highest elution peak.

SPR co-injection experiments showed that both YRLK and YKKD – unlike full-length 3G61 – were able to bind simultaneously with 3G124nc to GFP (Fig. 2(c)). The equilibrium dissociation constants (*K*_D_) determined by SPR of YRLK and YKKD towards GFP increased to 174 nM and 242 nM, respectively. This corresponds to an approximately 200-fold loss of affinity compared to 3G61, which has a *K*_D_ of 1.1 nM (Table 2, Fig. 1(e), SI Fig. 2)^14^. While this might suggest a significant contribution of binding through the N-cap, we show that the N-cap does not interact directly (see below), but contributes to stabilizing an interacting loop.

**Table 2:** Affinities and kinetics of GFP-binders.

Imobilized binding partner	Injected binding partner	k_a_ (M^−1^s^−1^)	k_d_ (s^−1^)	K_D_
GFP	3G124nc^a^	8.88 ± 0.01 × 10^5^	2.69 ± 0.006 × 10^−4^	303 pM
GFP	3G61^b^	7.61 ± 0.02 × 10^5^	7.97 ± 0.011 × 10^−4^	1.1 nM
GFP	YKKD^a^	1.98 ± 0.01 × 10^5^	4.81 ± 0.030 × 10^–2^	242 nM
GFP	YRLK^c^	2.42 ± 0.02 × 10^5^	4.22 ± 0.024 × 10^−2^	174 nM
GFP	gc_R7^c^	2.26 ± 0.005 × 10^5^	6.55 ± 0.045 × 10^−6^	29 pM
GFP	gc_R11^c^	2.22 ± 0.008 × 10^5^	8.40 ± 0.062 × 10^−6^	38 pM
GFP	gc_K7^c^	3.50 ± 0.008 × 10^5^	1.84 ± 0.007 × 10^−5^	52 pM
GFP	gc_K11^a^	4.06 ± 0.008 × 10^5^	1.16 ± 0.006 × 10^−5^	29 pM
GFP	nl_gc_R7^c^	7.59 ± 0.007 × 10^5^	8.26 ± 0.048 × 10^−6^	11 pM
GFP	3 × AF647_gc_R7^c, ^ ^d^	7.49 ± 0.006 × 10^5^	5.70 ± 0.064 × 10^−6^	8 pM
gc_R7	GFP^e^	7.32 ± 0.011 × 10^5^	1.08 ± 0.007 × 10^−5^	15 pM
gc_R7	eGFP^e^	5.67 ± 0.011 × 10^5^	9.25 ± 0.081 × 10^−6^	16 pM
gc_R7	sfGFP^e^	6.03 ± 0.011 × 10^5^	1.98 ± 0.008 × 10^−5^	33 pM
gc_R7	eYFP^e^	5.68 ± 0.012 × 10^5^	1.38 ± 0.009 × 10^−5^	24 pM
gc_R7	eCFP^e^	8.71 ± 0.012 × 10^5^	1.53 ± 0.007 × 10^−5^	18 pM
gc_R7	mCherry^e^	n.i.	n.i.	n.i.
gc_R7	mRuby^e^	n.i.	n.i.	n.i.

All data were measured by SPR. The statistical errors given are those obtained from the fits. n.i.: no apparent interaction. ^a^Sensogram depicted in Fig. 1(b). ^b^Data from Brauchle *et al*.^12,14^. ^c^Sensogram depicted in SI Fig. 2. ^d^This protein was triple-labelled with Alexa Fluor 647. ^e^Sensogram depicted in SI Fig. 4.

The *K*_D_ of 3G124nc (with the stabilized C-cap) did not change compared to 3G124 (with the old C-cap): *K*_D_s of 303 pM and 360 pM, respectively, were measured, and are thus within experimental error of the original constructs (Fig. 1(d), Table 2). Surprisingly, SEC-MALS revealed that 3G124nc had a dimeric fraction of approximately one third, in contrast to the original 3G124, which was predominantly monomeric (Fig. 3(a), Table 1)^14^.

Midpoints of thermal denaturation were assessed by circular dichroism measurements (222 nm) while heating from 20 to 92 °C (Fig. 3(b)). YRLK and YKKD had melting points of 66.0 °C and 66.6 °C, respectively. This was slightly lower than the values for two unselected DARPin library members with the same total number of repeats but a full N-cap^29^. 3G124nc did not show a clear transition up to 92 °C, a stability which is not unusual for full-length DARPins with three internal repeats and an optimized C-cap (Fig. 3(b), Table 1)^33^.

### Design and characterization of GFP clamps

To connect 3G124nc and either YKKD or YRLK we chose flexible GS-linkers. The linker length was estimated from the distance of the C-terminus of 3G124nc and the N-terminus of the truncated 3G61 in the structural superposition on GFP, which is 21 Å. A half-circle with this diameter would be spanned by approximately 10 amino acids (aa), assuming 3.3 Å per aa. Therefore, two linkers of 7 and 11 aa named GS7 (GGGSGGG) and GS11 (GGGSGGGSGGG) were tested for linking 3G124nc with either YKKD or YRLK. The resulting four constructs were named gc_K7, gc_K11, gc_R7 and gc_R11 according to whether they contained YKKD or YRLK and the linker used was 7 or 11 aa long. Additionally, a variant of gc_R7 devoid of lysines (nl_gc_R7; no lysine gc_R7) was designed. This construct can be functionalized or immobilized via N-hydroxysuccinimide (NHS) chemistry in a site-specific manner through introduction of defined lysines. All lysines, none of which were located at variable positions of the DARPin scaffold in gc_R7, were mutated to arginine, methionine or histidine (SI Fig. 1).

The five constructs were cloned and purified from *E. coli*. One litre of shake flask culture yielded about 100 mg of pure protein by a simple IMAC purification. SEC-MALS experiments showed symmetric elution peaks; in some cases very small additional peaks of higher molecular weight were observed (Fig. 3(a)). The measured molecular masses confirmed that all proteins were monomeric (Table 1).

Thermal denaturation of several GFP-clamps was assessed as described above; they show a transition between 68.7 °C and 71.8 °C. This probably corresponds to the melting of the truncated DARPin domain, whereas 3G124nc remains folded in the investigated temperature range (Fig. 3(b) and Table 1).

The affinity of all GFP-clamps was measured by SPR with GFP immobilized on the sensor surface. *K*_D_s between 29 pM and 52 pM were measured. The affinity of 11 pM for the lysine-free GFP-clamp is even slightly better than that of the other GFP-clamps. The association rate constants (*k*_a_) are between 2.22 × 10^5^ and 7.59 × 10^5^ M^−1^s^−1^, which are typical values for binding between two folded proteins. The dissociation rate constants, however, were very low (between 6.55 × 10^−6^ and 1.84 × 10^−5^ s^−1^) (Table 2). In other words, the longest half-life of dissociation is around 29 h.

The specificity of GFP-clamps was tested with different fluorescent proteins (FPs). For this purpose, we immobilized an *in vivo* biotinylated GFP-clamp (avi_gc_R7) on an SPR chip and injected GFP, eGFP^35,36^, superfolder GFP (sfGFP)^39^, enhanced yellow fluorescent protein (eYFP)^4^, enhanced cyan fluorescent protein (eCFP)^8,40^ and the red-fluorescent proteins mCherry^5^ and mRuby^6^ (sequences of all FPs are shown in SI Fig. 3). GFP, eGFP, sfGFP, eYPF and eCFP all showed very tight binding to the GFP-clamp with *K*_D_s between 15 pM (with GFP) and 33 pM (with sfGFP). Binding to these proteins was expected, since they have between 96% (sfGFP) and 99% (eGFP) sequence identity to GFP, which was used as the target in the ribosome display selection of the parental DARPins^14^. The affinity appears to be slightly better if the GFP-clamp is immobilized compared to the assay in which GFP is immobilized. This might be due to different accessibility of the immobilized binding partners or a consequence of experimental error. No interaction at all was detected with mCherry or mRuby, which is not surprising, since even though mCherry and mRuby also fold into an eleven-stranded β-barrel similar to GFP, the sequence identity with GFP is only around 30% for both proteins (SI Fig. 4, Table 2).

### Crystal structures of GFP-clamps

**Figure 4:**
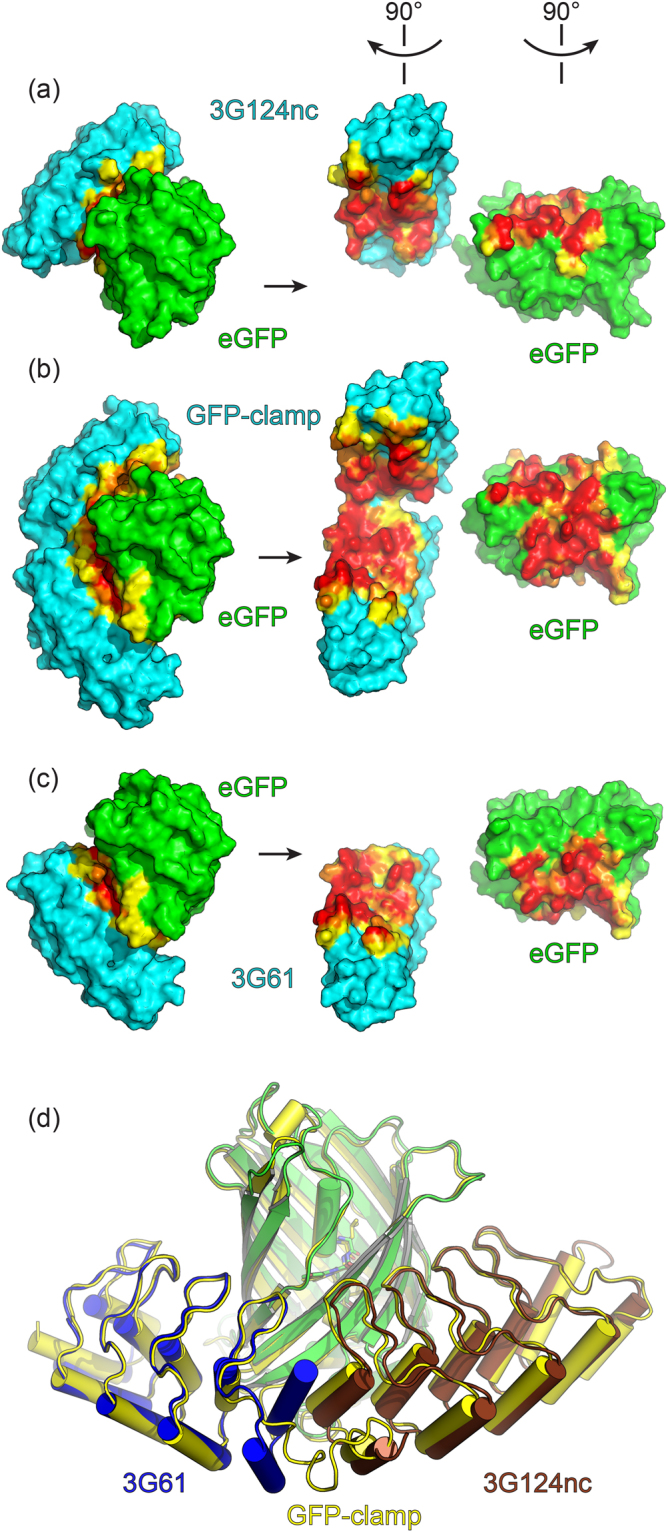
Crystal structures of GFP-clamps, as well as the individual DARPins 3G61 and 3G124nc, in complex with eGFP. (**a**) Complex between 3G124nc and eGFP; Complex is shown as found in the crystal structure on the left and in “open book” view on the right to show the binding interface. (**b**) Same representation as in (**a**) for the complex between a GFP-clamp (gc_K7) and eGFP. (**c**) Same representation as in (**a**) for the complex between 3G61 and eGFP. Interacting atoms in (**a**–**c**) are coloured according to their distance from the binding partner (red:<3.6 Å, orange: <5 Å, yellow: whole residues with some atoms closer than 5 Å). (**d**) Superposition of the parental DARPins and a representative experimental structure of a GFP-clamp (gc_K11).

To validate our design, the complex structures of eGFP with all GFP-clamps (gc_R7: PDB ID 5MAK; gc_R11 PDB ID 5MA3 and 5MA9; gc_K7 PDB ID 5MA4; and gc_K11 PDB ID 5MA5) were determined. The complexes crystallized under various conditions with different unit cells and one to four complexes per asymmetric unit (AU). All GFP-clamps form 1:1 complexes with eGFP, in which the clamp indeed wraps around approximately half the eGFP molecule and creates a continuous large interaction surface, similar to the superposition of the two parental DARPins on GFP (Fig. 4(a,b and c)). All GFP-clamps exhibit very similar topologies with typical Cα-RMSDs of 0.5 Å and overlay also well with the structures of parental DARPins 3G61 (PDB ID 5MAD) and 3G124nc (PDB ID 5MA6 and 5MA8) (Fig. 4(d)).

The complex interfaces were analysed with QtPISA v2.0.4^41^ and LigPlot+ ^42^. The buried surface area (BSA) of the GFP-clamp complexes is 1625 ± 72 Å^2^. Between 7 and 11 hydrogen bonds were found in the different structures. Hydrogen bonds of Trp79 from the GFP-clamp to Leu44 of eGFP, Trp79 to Leu220, Gln81 to Lys41, His114 to Gln204, Asp143 to Gln204, Asn156 to Arg73 and Phe226/230 (numbering for constructs with different linker lengths, respectively) to Asn198 seem to be the most crucial ones, since they are found in almost all complex structures. Besides hydrogen bonds, many hydrophobic interactions are found (SI Fig. 5). Interfaces in the 3G61:eGFP structure have a BSA of 755.3 ± 15.8 Å^2^, the interfaces of 3G124nc:eGFP account for 826.1 ± 13.2 Å^2^ of BSA. The sum of these two BSA equals 1617.5 Å^2^, which is almost identical to the average value obtained from the GFP-clamps, suggesting that no interface has been lost in the engineering of the clamp. All hydrogen bonds except two (GFP-residue Gln204 to Asp143 and His114 of eGFP) are also found in the complex structures of the parental DARPins with eGFP. The appearance of these additional hydrogen bonds in the GFP-clamp structures stems from a slightly altered conformation of the C-terminal end of 3G124nc, possibly caused by the crystallization conditions or the linkage to the truncated 3G61 domain.

The glycine-serine linkers between 3G124nc and the truncated 3G61 are not always resolved in the electron density and thus were not or only partially modelled in the majority of the structures. Whether the linker is visible or not largely depends on crystal contacts; e.g., the linker with the clearest electron density from the gc_K7:eGFP structure is stabilized by crystal contacts with a symmetry-related GFP-clamp molecule. All linkers that are fully or partially modelled exhibit elevated B-factors compared to the rest of the GFP-clamp if they are not stabilized by direct crystal contacts.

### Use of GFP-clamps as immobilization agent in SPR experiments

GFP-clamps are very useful for oriented immobilization of GFP-tagged proteins on SPR sensor chips due to their very slow dissociation rates. This strategy may circumvent much of the experimentation in covalently immobilizing a target. Furthermore, the establishment of target regeneration methods can be circumvented — an issue especially for labile targets — if the whole target-GFP complex can be eluted at the end of the cycle. This, however, requires that a generic regeneration strategy of such chips can be devised that can separate GFP from the clamp despite its very tight binding.

As a proof-of-principle, we injected 20 nM GFP over a neutravidin chip that was coated with avi_gc_R7. After 10 min, the chip was regenerated with two injections of 1 M glycine, pH 2.0 for 30 s. This cycle was repeated ten times to estimate the stability of such a surface over time. The binding signal of the tenth GFP injection remained almost unchanged; only a decrease of about 5% in binding capacity was observed (Fig. 3(c)).

### Protein purification with a GFP affinity-column

**Figure 5:**
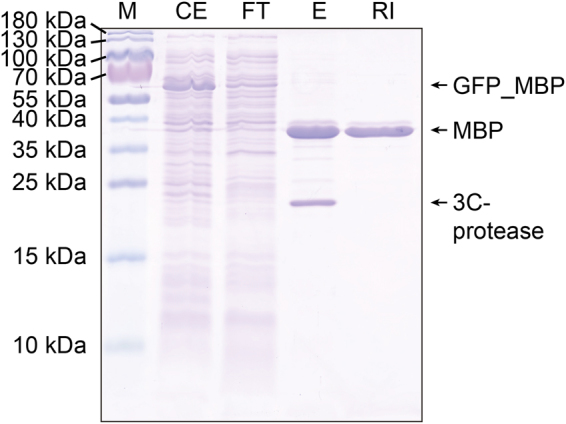
Protein purification using GFP-clamps. A fusion protein between sfGFP and maltose binding protein (GFP_MBP) is purified from crude *E. coli* extract with a GFP affinity resin. M: Protein size marker; CE: crude extract; FT: flow-through, E: elution with 3C-protease; RI: purified MBP after removal of His-tagged 3C-protease by reverse IMAC. The gel image was recorded from a single gel.

A triple lysine tag was added to the N-terminus of a lysine-free GFP clamp (KKK_nl_gc_R7, SI Fig. 1), and coupled to NHS-activated Sepharose beads to produce an inexpensive GFP affinity resin. We used an sfGFP fusion to maltose binding protein (GFP_MBP) as a model to show that GFP-tagged proteins can be specifically isolated from crude *E. coli* extracts. The fusion protein was efficiently captured from the crude extract. After washing, MBP was eluted by 3C-protease cleavage of the 3C recognition site introduced in between sfGFP and MBP. His-tagged 3C-protease was removed by an additional reverse IMAC step. MBP purified with this protocol was very pure judging from SDS-PAGE analysis (>95%) (Fig. 5). After regeneration with 6 M Gdn HCl, 20 mM glycine, pH 1.5 to remove the bound sfGFP our resin could be reused several times.

### High-sensitivity detection of GFP-tagged proteins on the surface of mammalian cells

We generated GFP-clamp versions with a defined number of labelling sites by introducing one to three cysteines into the gc_R7 sequence (3 × cys_gc_R7, sequence in SI Fig. 1). Coupling of up to three Alexa Fluor 647 molecules per GFP-clamp via maleimide chemistry was straightforward and very efficient (SI Methods), resulting in the triple-labelled construct 3 × AF647_gc_R7. The binding affinity of dye-labelled GFP-clamps for GFP remained essentially unaltered (Table 2, SI Fig. 2).

**Figure 6:**
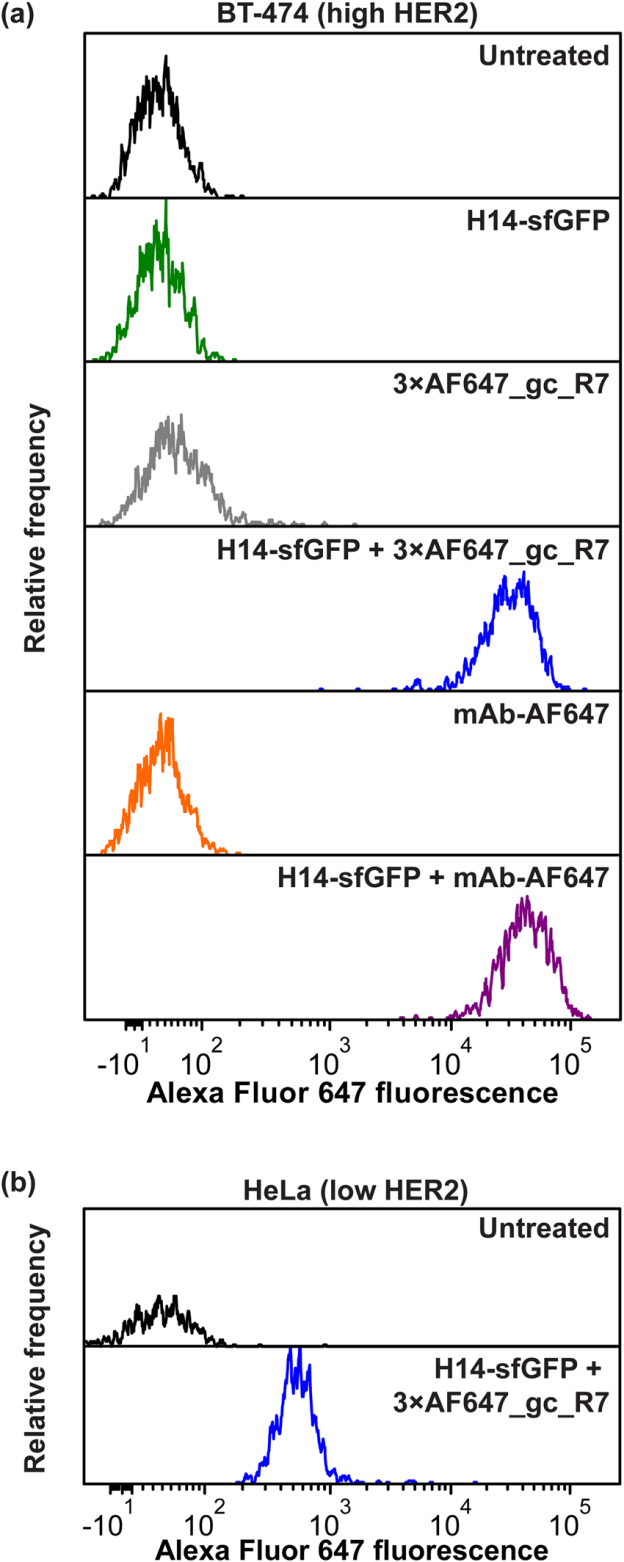
GFP-clamp as monovalent tight-binding secondary detection reagent with multiple labels in flow cytometry. (**a**) HER2-binding H14-sfGFP can be specifically detected on the surface of the HER2-overexpressing breast cancer cell line BT-474 using a triple AF647-labelled GFP-clamp. The performance is similar as with a commercially available rat monoclonal FM264G, which however is bivalent and thus may crosslink GFP, as well as the bound receptor, possibly leading to unintended biological effects. (**b**) Robust detection is also possible on HeLa cells, even though they express only low levels of HER2.

We then explored the potential of fluorescently labelled GFP-clamps as a monovalent secondary detection reagent. We employed DARPin H14^43,44^ fused to sfGFP (H14-sfGFP) as primary reagent to detect the oncoprotein HER2 expressed on the surface of mammalian cells. When used as secondary detection reagent on BT-474 breast cancer cells, which strongly overexpress HER2, the labelled GFP-clamps resulted in a specific signal, very similar to that of a commercially available rat monoclonal antibody (FM264G) (Fig. 6(a)), which, however, may crosslink GFP and thus also the bound receptor and may thus elicit unintended biological consequences. We were also able to obtain a robust signal on the surface of HeLa cells of cervical cancer origin, which have been described as HER2-negative by histology^45^ and express only low levels of HER2^46^ (Fig. 6(b)).

### Pull-down experiments

**Figure 7:**
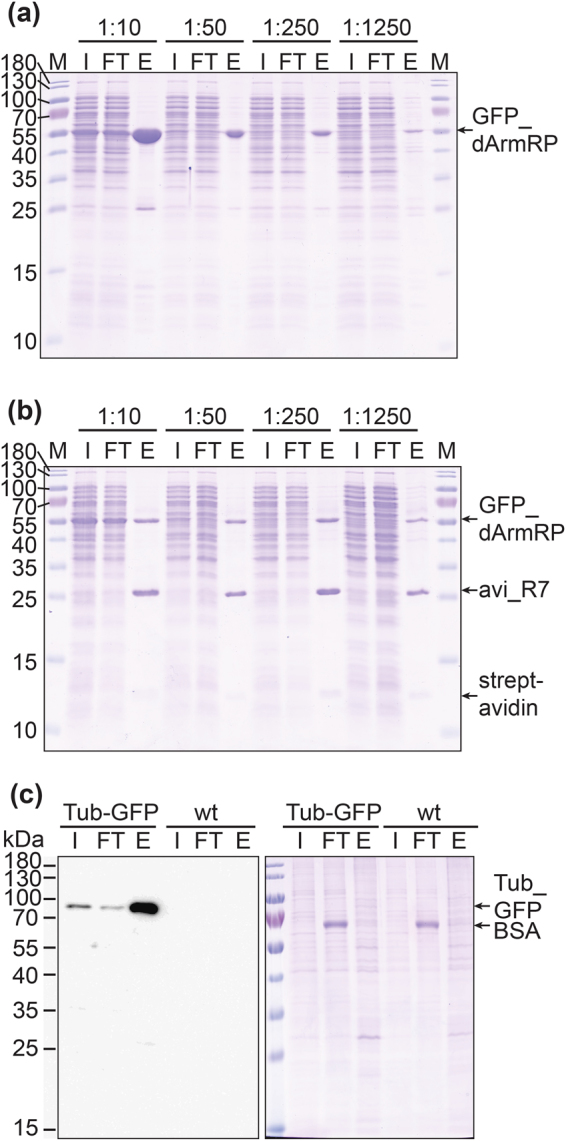
Pull-down experiments with GFP clamps. (**a**) *E. coli* extract expressing GFP_dArmRP was diluted with crude extract from a non-expressing strain (from 1:10 to 1:1250). Pull-downs were performed with lysine-free GFP-clamps immobilized via added lysines on NHS-Sepharose beads and analysed by Coomassie staining. (**b**) Same experiment as in (**a**), but pull-down was performed with *in vivo* biotinylated GFP-clamps and magnetic streptavidin beads. (**c**) Pull-down experiments with crude extracts from HeLa cells stably expressing a tubulin-GFP fusion (Tub-GFP) and ‘wild type’ HeLa cells (wt). Analysis by western blotting on the left and Coomassie staining on the right. M: Protein size marker; I: input (crude extracts); FT: flow through (crude extract after pull-down); E: elution (beads loaded on the gel). Panels (a) and (b) depict single Coomassie-stained gels, panel (c) depicts a single blot image (left) and a single Coomassie-stained gel (right).

Pull-down experiments with GFP-tagged proteins were carried out in two different formats. First, 20 µl of the GFP-clamp functionalized Sepharose beads (see above) were used and second, 10 µg of biotinylated GFP-clamp and streptavidin-coated magnetic beads were employed to capture GFP-fusions. In the first experiment, we tested both methods to see if they can specifically enrich a fusion between sfGFP and a designed armadillo repeat protein (sfGFP_dArmRP)^47^ from *E. coli* crude extracts. To test whether this is possible even if the target protein is not abundantly present, the crude extract was diluted with crude extract from a non-expressing *E. coli* strain (Fig. 7(a) and (b)). The GFP-clamp functionalized Sepharose beads were not saturated, and the amount of fusion protein that was pulled down scales well with its concentration in the dilution of the crude extract. In the magnetic bead set-up, the amount of GFP-clamp on the beads seems to be limiting, since roughly the same amount of sfGFP_dArmRP was enriched from all dilutions except the highest one where sfGFP_dArmRP appears to be limiting. Boiling the Sepharose beads in SDS loading buffer should only elute the GFP_dArmRP band. The magnetic beads would also release GFP-clamps and streptavidin, since they are not covalently coupled to the beads. All these bands were indeed visible. However, the Sepharose beads also seemed to pull down some additional unspecific bands, resulting in a less pure elution fraction. In the lower dilutions, also an enriched band of approximately 25 kDa was visible. This is most probably GFP that was cleaved off by endogenous *E. coli* proteases.

Finally, pull-down experiments with crude extracts of HeLa cells stably expressing tubulin-GFP fusions (Tub-GFP) and ‘wild-type’ (wt) HeLa cells were performed using GFP-clamps coupled to Sepharose beads. Here, the enriched Tub-GFP band was not visible by Coomassie stain analysis (Fig. 7(c) right panel). However, when western blotting was used for quantification, it became clear that the target protein was massively enriched after the pull-down and only traces of Tub_GFP remained in the supernatant. As expected with the wt cell line, no bands were visible in the western blot (Fig. 7(c) left panel).

## Discussion

The structure-guided design approach of a GFP clamp, which required a truncation of the N-cap of DARPin 3G61, attests to the robustness of the DARPin scaffold. Such a truncation, allowing the linking of binders with overlapping epitopes, is only possible with repeat protein scaffolds. In such designed proteins, single repeats can be removed to avoid clashes, because in these scaffolds intramolecular interactions are formed between residues within a repeat that are close in primary sequence, and interactions between repeats are identical. Removing a terminal repeat and thus exposing a repeat interface to the solvent requires to make the surface polar, however. This could be achieved by only four point mutations that restored monomeric behaviour and high stability (Table 1) in the resulting construct, thereby accommodating the truncation. These point mutations are probably generically applicable to stabilize truncated DARPins, since every exposed internal repeat interface will be the same. This approach is therefore different from the random generation of multivalent binding proteins that are linked by flexible linkers (see, e.g., ref.^48^), in that the approach here uses a structure-guided approach from the knowledge of crystal structures of complexes, where even overlapping epitopes can be accommodated.

Upon truncation the *K*_D_ increased approximately 200-fold (Table 2). Since the 3G61:eGFP structure suggests that the N-cap itself does not directly contribute to the interaction, we believe that the loss in affinity is seen because the first β-loop of the truncated DARPins is no longer anchored by the N-cap. In solution, this loop might thus be disordered and only fold upon binding, with an entropic penalty. Even though their molecular weight is smaller, truncated DARPins show a lower retention volume than 3G124nc in SEC (Table 1). This might be caused by this unfolded β-loop in solution. The affinity loss might be overcome by an affinity maturation of the truncated DARPins, as described previously^49–52^, but this was not done in the present study, since clamps with low picomolar *K*_D_ could be readily obtained.

The structures of the GFP-clamps confirm the intended design: both DARPin-derived domains overlay well with the parental DARPins when superimposed on eGFP (Fig. 4(d)). Most interactions between the DARPins and eGFP are found in the single DARPin complexes as well as in the GFP-clamp complexes, confirming that both domains can bind their respective epitope without strain or steric hindrance.

Depending on the set-up of the SPR assays, the GFP-clamps reached affinities between 11 pM and 52 pM against GFP and closely-related FPs (Table 2). These are among the tightest interactions of single-chain binding molecules to GFP described so far. Single nanobodies achieve affinities down to 450 pM^25,26^ and linked nanobodies binding different epitopes had affinities of 268–36 pM^25^. For αRep binders affinities to eGFP between 1.4 nM and 14 nM were shown^12^.

Besides their high affinities, the described GFP-clamps also offer very good expression yields in *E. coli* of ca. 100 mg/L and excellent biophysical properties like high melting points, good stability at low pH and monomeric behavior, which allows their straightforward use in different applications. With the development of the lysine-free GFP-clamp, which like all DARPins is also devoid of cysteine residues, site-specific immobilization or functionalization via NHS-chemistry and maleimide chemistry is possible. Incorporation of azidohomoalanine as a methionine surrogate has been established for DARPins, allowing functionalization via click chemistry^53,54^, although this requires replacing two internal methionines, as has been done before^55^. Therefore, when using all three methods at once, even an orthogonal site-directed triple functionalization becomes possible.

Capture techniques for ligands on SPR chips are popular, since in contrast to typical random covalent coupling techniques, the ligand is immobilized in a specific orientation and thus a more homogeneous sensor surface is produced^56^. Slow dissociation rates of the capturing agent are required, because this rate limits the affinity range of interactions that can be studied. With a *k*_d_ of around 1 × 10^−5^ s^−1^, the GFP-clamps are well suited for such an approach. Furthermore, GFP-clamps can easily be produced in an *in vivo* biotinylated form for use with commercially available neutravidin or streptavidin chips. Alternatively, carboxymethylated SPR chips can be functionalized by coupling to defined lysines added to an otherwise lysine-free GFP-clamp; usually higher ligand surface densities are obtained with this approach than with neutravidin/streptavidin chips. We showed that the GFP-clamp:GFP interaction can be regenerated repeatedly by injections of glycine, pH 2.0, and that upon neutralization the protein completely refolds such that the binding capacity of the sensor surface is almost unaffected by this treatment. In practice, this regeneration strategy can be used to exchange or renew the GFP-tagged ligand several times, which allows a labile ligand to be exchanged without establishing regeneration methods, and in general more experiments can be run on the same sensor chip (Fig. 3(c)).

With our GFP affinity-resin we were able to obtain pure protein in a two-step column purification. Such resins are of great interest since many proteins are expressed as fusions with GFP or closely related proteins. So far, similar resins have been described using GFP-specific antibodies^57^ or nanobodies^11^. However, to our knowledge, these have not become very widely used, probably due to low expression yields of these reagents, restricting an inexpensive large-scale production of the resins. GFP-clamps, on the other hand, exhibit very high expression yields of typically 100 mg/L in *E. coli*, allowing for efficient and low-cost production of affinity resin even on large scales. Also, the high affinity of the GFP-clamps permits very stringent washing. The lysine-free GFP-clamp is advantageous, since it can be coupled to NHS-activated Sepharose beads in a site-directed manner exploiting the engineered “lysine tag”. A column produced with GFP-clamps coupled randomly via the lysines of the scaffold was leaky for GFP, meaning that some GFP fusion proteins eluted already during washing steps (data not shown); this phenomenon was not observed with the resin produced with lysine-free GFP-clamps. We believe this happens because, in the case of random coupling, both domains of the GFP-clamp are coupled to the beads and hence are not mobile enough to bind GFP simultaneously, resulting in a lower affinity.

Elution of the protein of interest was performed by 3C-protease cleavage at an engineered cleavage site between the protein of interest and GFP: this was the only way to elute proteins under mild conditions, since breaking the GFP-clamp:GFP interaction requires very low pH (pH < 2), which in most cases will be detrimental to the protein of interest. An advantage of this elution technique is that another layer of specificity is applied to the purification, since only proteins with a 3C-protease recognition site will be eluted. The His-tagged 3C-protease is then removed by a second chromatography step, ideally an IMAC, with the protein of interest running in the flow-through. It should be pointed out that all affinity-based anti-GFP resin columns described so far lack a generic and mild elution strategy for full-length proteins^11,57^. The high stability of GFP-clamps allows one to strip GFP from the column after purification and even reuse this inexpensive resin, making even very large scale purifications attractive.

GFP fusions are universally employed to study proteins by microscopy and flow cytometry, but labelled antibodies are frequently required as secondary detection reagents to achieve signal amplification. However, crosslinking by multivalent affinity reagents may alter the apparent biological activity of the protein of interest. For quantitation, the signal observed may often not be directly proportional to the amount of GFP present, because avidities of the bivalent immunoglobulins depend on the local density of the antigen^58^. Furthermore, the typical coupling of fluorophores to primary amines has to be optimized for each case in order to balance desired high fluorescence intensity and potential interference with binding^59^, and avoid self-quenching by homo-FRET in case of overlabelling^60^. Immunoglobulins are therefore hardly suitable detection reagents to study the binding thermodynamics or kinetics of GFP-tagged proteins.

GFP-clamps are interesting generic affinity reagents for such applications, because they provide virtually free choice of the fluorophore to be coupled, and full control over labelling stoichiometry and site, because insertion of cysteines enables specific maleimide coupling. As therefore expected, we did not observe deleterious effects of triple-labelling on the affinity of GFP-clamps (SI Fig. 2). Also, due to its excellent affinity, no multimerization is required, which will more likely result in a linear signal dependence on the amount of antigen, and no crosslinking.

We demonstrate here that a triple-labelled variant of the GFP-clamp is useful as a secondary detection reagent in flow cytometry, which performs, as a monovalent entity, very similar to a commercially available antibody, which even carries more fluorophores, but would, because of its bivalent nature, crosslink GFP and thus the bound receptor (Fig. 6) and potentially might elicit unwanted biological effects.

Pull-down experiments were also performed successfully with our GFP-clamps. Similar experiments have also been described for other GFP binders^11,12,25^; again, we see the advantage of our GFP-clamps in their high affinity, easy production and stability. This results in highly specific and sensitive, inexpensive and long-term stable reagents. Furthermore, they are versatile, since all common immobilization chemistries (NHS, maleimide, biotin-avidin and click-chemistry^53–55^) can be used.

While the reagents show high specificity in all assays including pull-downs, after permeabilization of cellular membranes by detergents, we had previously observed some cell-line independent background binding in flow cytometry and microscopy. For these applications, we currently recommend use of the GFP-clamp for cell surface targets, and not for intracellular targets, where permeabilization is needed.

In summary, the design of a clamp from two DARPins with overlapping epitopes exploits the robustness of this scaffold, and has led to molecules with very useful properties in the case of GFP binders.

## Methods

### Cloning

All cloning steps were performed with restriction enzymes from New England Biolabs (NEB) according to the manufacturer’s instructions. Phusion High Fidelity DNA Polymerase (NEB) was used for PCRs according to the manufacturer’s instructions, with oligonucleotides purchased from Microsynth or Integrated DNA Technology (IDT). *E. coli* strain XL1 Blue (Stratagene) was used for all cloning steps. Coding sequences for 3G124nc, nl_gc_R7 and part of 3G61 were ordered as gBlock gene fragments from IDT. Truncation mutants YKKD, YRLK and YRID were assembled from overlapping oligonucleotides and a gBlock gene fragment by PCR. GFP-clamps were assembled by PCR with oligonucleotides encoding either GS7 or GS11 linkers. FPs were PCR-amplified from different vectors and subcloned. All gene constructs were cloned into pQIq-based vectors^55^ carrying the respective N- or C-terminal tags.

### Protein expression

Proteins were expressed in the *E. coli* strain XL1 Blue (Stratagene). 2xYT medium containing 100 µg/ml ampicillin and 0.5% glucose was inoculated to an OD_600_ of ca. 0.15 from an overnight culture (2xYT, 100 µg/ml ampicillin and 1% glucose) and grown at 37 °C. Expression was induced with 500 µM IPTG when the OD_600_ reached ca. 0.7 and continued for 5 h at 37 °C or overnight at 30 °C. For producing *in vivo* biotinylated proteins, *E. coli* was co-transformed with the plasmid pBirAcm (Avidity Inc.), and 35 µg/ml chloramphenicol was used as a selection marker in all media. Prior to induction, 50 µM of biotin was added to expression media. Expression cultures were pelleted by centrifugation (5 min, 5000 *g*) and resuspended in 25 ml of TBS_W (50 mM Tris-HCl pH 8, 400 mM NaCl, 20 mM imidazole and 10% glycerol) per litre of expression culture and either directly processed further or frozen at −20 °C.

### Protein purification

Cells were lysed by a passage through a French press system and sonication. Cell debris was removed by centrifugation (20 min, 25,000 *g*). Crude extracts were applied to IMAC columns (Ni-NTA Superflow resin, Qiagen) and washed with 10 column volumes (CV) of TBS_W, 10 CV of high-salt buffer (50 mM Tris-HCl pH 8, 1000 mM NaCl, 20 mM imidazole), 10 CV of low-salt buffer (50 mM Tris-HCl pH 8, 20 mM NaCl, 20 mM imidazole and 20% glycerol) and again 10 CV of TBS_W. Proteins were eluted with TBS_E (same as TBS_W but containing 350 mM imidazole). Proteins carrying a His-tag that could be removed by 3C-protease were cleaved by adding 2% w/w of 3C-protease while dialyzing against PBS overnight whereas non-cleavable proteins were dialyzed against PBS. Uncleaved proteins and 3C-protease were removed by reverse IMAC.

For crystallization, complexes of eGFP and GFP-binders were isolated by SEC on a HiLoad 16/600 Superdex 200 pg column (GE Healthcare) with 10 mM Tris/HCl, pH 7.4 and 100 mM NaCl as running buffer and concentrated to 20 mg/ml.

### Size exclusion chromatography multi-angle light scattering (SEC-MALS)

SEC-MALS experiments were run on an Agilent LC1100 chromatography system (Agilent Technologies) coupled to an Optilab rEX refractometer (Wyatt Technology) and a miniDAWN three-angle light-scattering detector (Wyatt Technology). A 24 ml Superdex 200 10/30 column (GE Healthcare Biosciences) was used with PBS as running buffer. 50 µl of protein samples at 1–1.5 mg/ml were injected. ASTRA software (version 6.0.1.10; Wyatt Technology) was used for analysis.

### Circular dichroism

Circular dichroism (CD) measurements were performed on a Jasco J-810 instrument (Jasco) using 20 μM protein and a 0.5 mm path length cylindrical thermo-cuvette. Heat denaturation curves were collected by observing the CD signal at 222 nm in the temperature range from 20 to 92 °C (data pitch: 0.2 °C, heating rate: 1 °C/min). The mean residual ellipticity (MRE) (blank corrected) was calculated and normalized by setting the initial values (20 °C, folded) as 0 and the putative completely unfolded protein (MRE = 0) as 1, resulting in the unfolded fraction (f_u_). Data were fitted to a two-state unfolding model with sloping baselines^61,62^ with eqs 1 and 2. Since the full reversibility and two-state nature of this system is questionable, all fits were only used to estimate the midpoint of thermal denaturation and not any other parameters:

1 $${f}_{u}=\frac{1}{1+\frac{1}{{e}^{\frac{{\rm{\Delta }}G}{RT}}}}\times ({y}_{l}+{m}_{l}\times T-{y}_{u}-{m}_{u}\times T)+{y}_{u}+{m}_{u}\times T$$

2 $${\rm{\Delta }}G=\frac{{T}_{m}-T}{{T}_{m}}\times {\rm{\Delta }}H-({T}_{m}-T)\times {\rm{\Delta }}{C}_{p}+T\times {\rm{\Delta }}{C}_{p}\times ln\frac{{T}_{m}}{T}$$

ΔG and ΔH: free energy and enthalpy of unfolding, respectively; T_m_: midpoint of thermal denaturation; T: temperature; ΔC_p_: change in heat capacity at constant pressure; y_l_ and y_u_: y-axis intercepts of the lower and upper baseline, respectively; m_l_ and m_u_: slopes of the lower and upper baseline, respectively.

### Crystal screening

Sitting-drop vapor-diffusion experiments were performed using a Phoenix crystallization robot (Art Robbins Instruments). Sparse-matrix screens from Hampton Research and Molecular Dimensions in 3-Drop Intelli-Plate 96-3 LVR (Hampton) at 4 °C were used for screening of crystallization conditions. Protein solutions were mixed with reservoir solutions at 1:1, 1:2 or 2:1 ratios (400–450 nl final volume) and equilibrated against 70 μl of reservoir solution. Well diffracting crystals of 3G61:eGFP were obtained with a fine screen (PEG and pH gradient) and stroke seeding from crushed initial crystals.

### Diffraction data collection

Single crystals were picked from drops as cryo-protectant 5–15% (v/v) of ethylene glycol was added to the mother liquor and crystals were submerged in it for several seconds prior to flash-freezing in liquid nitrogen. Crystals for the complex structures of 3G61 and gc_R7 were directly frozen in mother liquor. Data were collected using a Pilatus detector system (Dectris Ltd) on beam line X06DA or X06SA at the Swiss Light Source (Paul Scherrer Institute, Villigen, Switzerland).

### Structure determination

Diffraction data were processed using programs XDS, XSCALE and XDSCONV^63^. The crystal structures were solved by molecular replacement with PHASER^64^. PDB IDs: 1GFL (GFP)^65^ and 4DUI (DARPin) were used as search models for the structures of gc_R7:eGFP and 3G124nc:eGFP; for all other structures the gc_R7:eGFP structure was used as search model. Structure refinement was done with REFMAC5^66,67^ and Phenix-Refine^68,69^, model building was done in COOT^70,71^. Five percent of data were set aside to calculate the *R*_*free*_ value. Figures were prepared in PyMOL (available from www.pymol.org). Crystallization conditions, data collection and refinement statistics are summarized in SI Table 1.

### SPR measurements

All SPR experiments were performed on a ProteOn XPR36 instrument on a NLC chip (Biorad) in PBS containing 0.005% Tween-20. Two ligand channels were coated with either *in vivo* biotinylated GFP or avi_gc_R7. A kinetic titration approach^72^ was used; five increasing concentrations of the same DARPin, GFP-clamp or FP were injected consecutively over two measuring spots (duplicates) for 5 or 6 min, followed by a dissociation phase of 15 min without regeneration at the end, then the next higher concentration was injected. The dissociation phase of the highest analyte concentration was extended to 2.5–3 h. Data were double-referenced in the ProteOn manager software. Datasets were fitted in the BiaEvaluation software to a kinetic titration model^72^. The truncated DARPins YKKD and YRLK dissociated completely within 15 min, hence all injections were overlaid and fitted to a classical Langmuir model in the ProteOn manager software.

For the co-injection experiments, GFP was immobilized in one ligand channel; the first injection was 1.5 µM of 3G124nc, the second injection contained a mixture of 1.5 µM 3G124nc and 1.5 µM of 3G61, YKKD or YRLK. Data were referenced in the ProteOn manager software and inspected manually.

The regeneration test was done by repeated injection of 20 nM GFP over six avi_gc_R7-coated measuring spots and two regeneration steps (each 30 s, 1 M glycine pH 2.0). Data of single injections were aligned to baseline prior to injection and then concatenated.

### Preparation of GFP-affinity column

NHS-activated Sepharose 4 Fast Flow beads (GE Healthcare, 8 ml of slurry) were washed with 40 ml of 0.01 M HCl. Fifty milligrams of purified KKK_nl_gc_R7 in PBS (SI Fig. 1) were added and incubated for 2 h at RT. Absorbance of the flow-through at 280 nm was measured and showed that all protein had been coupled to the beads. Beads were washed with PBS and stored in PBS with 1 mM sodium azide at 4 °C where they were stable for several months.

### Protein purification using a GFP-affinity column

For protein purification 100 µl of beads were packed to a column and equilibrated with 5 ml TBS (50 mM Tris/HCl, pH 7.5, 150 mM NaCl). Crude extract of GFP_MBP-producing *E. coli* was loaded onto the column. Subsequently, the column material was washed two times with 1 ml of TBS_W (without imidazole) and once with TBS high salt (50 mM Tris/HCl, pH 7.5, 1000 mM NaCl). MBP was eluted by cleavage with 3C-protease (3 h at RT). 3C-Protease was removed from the elution fraction by reverse IMAC. The resin was regenerated twice with 1 ml of 6 M Gdn-HCl, 20 mM glycine, pH 1.5, to remove sfGFP and the resin could be reused several times.

### Pull-down assays

A crude extract of *E. coli* expressing GFP_dArmRP was mixed at different ratios with a crude extract from non-expressing *E. coli* cells and diluted 5-fold in PBS. Crude extracts of HeLa cells (wt or expressing a GFP-tubulin fusion) were prepared by lysis of 1.5 × 10^6^ cells in 100 µl of lysis buffer (20 mM Tris/HCl pH 7.5, 150 mM NaCl, 0.5 mM EDTA, 24 µg/ml 4-(2-aminoethyl) benzenesulfonylfluoride, 0.5% Nonidet P-40) for 30 min and centrifuging for removal of debris and aggregates (20,000 *g*, 15 min). Supernatants were diluted 5-fold in PBS. Crude extracts (500 µl) were added to 20 µl of nl_gc_R7-functionalized Sepharose beads (via a lysine tag, see above) and incubated for 1 h at RT while shaking. After centrifugation (1 min at 5000 *g* at 4 °C), the supernatant was removed. The bead pellet was washed two times in 1 ml of TBS and once in TBS high salt (50 mM Tris/HCl, pH 7.5, 1000 mM NaCl). After the last washing step, the beads were resuspended in SDS sample buffer and boiled for 10 min at 95 °C and loaded on a polyacrylamide gel. The beads used for the experiment with HeLa extract were pre-incubated in TBS with 0.5% bovine serum albumin (BSA) for blocking, centrifuged, and blocking buffer was removed.

Pull-down assays with avi_gc_R7 were carried out by adding 10 µg of avi_gc_R7 to crude extracts and incubation for 30 min. Then 20 µl of streptavidin-coupled Dynabeads (ThermoFisher Scientific) were added and incubated for another 30 min. Beads were pelleted by magnets instead of centrifugation; otherwise the procedure was identical.

### Western blotting

Western blotting analysis was performed by wet blotting of the SDS-gel to a PVDF membrane (Millipore) in blotting buffer (20 mM Tris–HCl (pH 8.3), 150 mM glycine, 0.02% (w/v) SDS and 20% (v/v) methanol) for 1 h at 100 V. Membranes were blocked with casein blocking buffer (Sigma) for 45 min at RT on a roller mixer. A polyclonal rabbit anti-GFP antibody (Rockland Immunochemicals Inc., 1:4000 in casein buffer) and a secondary goat anti-rabbit IgG antibody horseradish peroxidase conjugate (Sigma, 1:10,000 in casein buffer) were used for detection. Each antibody was applied for 45 min on a roller mixer, after both antibody steps the membrane was washed 4 times for 5 min with PBS-T (PBS with 0.05% Tween 20). Chemiluminescence was recorded on a Fuji-Film LAS-3000 device using SuperSignal West Pico Chemiluminescent Substrate (ThermoFisher Scientific).

### Accession numbers

The atomic coordinates and structure factors of all DARPin:eGFP and GFP-clamp:eGFP complex structures have been deposited in the PDB (PDB ID: 5MA3, 5MA4, 5MA5, 5MA6, 5MA8, 5MA9, 5MAD and 5MAK).

## Electronic supplementary material

Supplementary Information
