# Supplementary material for: Design and applications of a clamp for Green Fluorescent Protein with picomolar affinity
Source: Sci Rep. 2017 Nov 24;7:16292. doi: 10.1038/s41598-017-15711-z (PMC5701241; doi:10.1038/s41598-017-15711-z)
Supplement: Supplementary file 1 — Supplementary Information [file 41598_2017_15711_MOESM1_ESM.pdf]

Supplementary Information

for

Design and applications of a clamp for Green Fluorescent Protein with picomolar affinity

Simon Hansen[a, 1], Jakob Stüber[a], Patrick Ernst[a], Alexander Koch[a], Daniel Bojar[a, 2], Alexander Batyuk[a, 3], Andreas Plückthun[a, *]

[a] Department of Biochemistry, University Zürich, Winterthurerstrasse 190, 8057 Zürich, Switzerland

[1] Present address: Department of Early Discovery Biochemistry, Genentech, 1 DNA Way, South San Francisco, California, 94080, USA

[2] Present address: Department of Biosystems Science and Engineering, ETH Zürich, Mattenstrasse 26, 4058 Basel, Switzerland

[3] Present address: Linac Coherent Light Source, SLAC National Accelerator Laboratory, 2575 Sand Hill Road, Menlo Park, California 94025, USA

Correspondence:

*Andreas Plückthun
Tel. +41-44-635 5570, Fax. +41-44-635 5712
e-mail: plueckthun@bioc.uzh.ch

SI Methods

Fluorescent labeling of GFP-clamps

One to three cysteine residues were added to gc_R7. N- or C-terminal cysteines were added to the gc_R7 ORF by subcloning into vectors that contained the respective cysteine-tags. The serine residue within the GS7-linker was mutated to cysteine by site directed mutagenesis (sequences and nomenclature in SI Fig. 1). Cys-containing proteins were expressed and purified as described in the main text, but all buffers contained 10 mM β-mercaptoethanol and no dialysis was performed. After purification, the buffer was exchanged to PB (15 mM phosphate, pH 7.4) with 30 mM TCEP using illustra NAP-5 columns (GE Healthcare), and incubated at RT for 45 min. A second buffer exchange to PB (without TCEP) in a N_2-atmosphere was performed. gc_R7-constructs (500 µl, 50-100 µM) were mixed with a 2-fold molar excess (over cysteine residues) of Alexa Fluor 647 C2 Maleimide (ThermoFisher Scientific, 10 mM in acetonitrile) in a N_2-atmosphere and incubated overnight at RT. Remaining free dye was quenched by adding DTT (30 mM, 30 min incubation) and then removed by buffer-exchange to PBS (NAP-5 column) and subsequent dialysis in PBS overnight.

The degree of labeling was calculated from the absorbance at 280 nm (taking into account the absorption of the dye) and at the absorbance at the extinction maximum of the dye. Extinction coefficients were provided by the manufacturer of the dye or calculated from the sequence of the protein. In some cases, the degree of labeling was confirmed by mass spectrometry. In all cases a nearly complete labeling (>95%) of all cysteines was obtained with the reaction conditions given above (data not shown). The affinity of the construct 3×AF647_gc_R7 (3×cys_gc_R7 triple-labeled with Alexa Fluor 647) was tested with SPR and was not decreased compared to unlabeled gc_R7 (SI Fig. 2).

Flow cytometry

Subconfluent BT-474 or HeLa cells (ATCC) were harvested by trypsinization, and resuspended to a concentration of 3.4×10^5 cells ml^{-1} in Dulbecco's phosphate buffered saline (DPBS) supplemented with metabolic inhibitors, 50 mM sodium azide and 10 mM 2-deoxy-D-glucose, to yield PBSA50D, and the cells inactivated by incubating for 30 min – as for all following steps, at room temperature. Then, H14-sfGFP was added to a final concentration of 100 nM to the respective 1 ml cell suspension aliquots, and incubated for a further 30 min. Cells were washed twice by pelleting (800 g, 1 min for this and all following centrifugation

steps) and resuspending in 1 ml DPBS. Afterwards, cells were resuspended in solutions of either 50 nM 3×AF647_gc_R7, the rat monoclonal antibody FM264G labeled with Alexa Fluor 647 (BioLegend) diluted 1:200 in PBSA50D, or buffer alone, and incubated for 20 min. After washing twice with 1 ml DPBS, cell pellets were resuspended and incubated for 15 min in a LIVE/DEAD Fixable Aqua Dead Cell Stain solution (Invitrogen) in DPBS, prepared according to the instructions of the manufacturer. Finally, cells were washed once in 1 ml PBSBA (DPBS supplemented with 1% (w/v) bovine serum albumin and 0.1% (w/v) sodium azide) and once in 1 ml DPBS, and resuspended in 0.5 ml DPBS for measurement on a LSR Fortessa II (BD) flow cytometer. Data were analyzed using FCS Express 5 Flow 5.01.0082 (De Novo Software), restricting analysis to live (non-permeabilized) cells according to the L/D stain and singlets.

SI Figures

```
N-terminal Tags:
              MRGS_His6: M R G S H H H H H H G S
          MRGS_His6_GCG: . . . . . . . . . . G C G G S
          MRGS_His10_3C: . . . . . . . . . . H H H H G G G S L E V L F Q|G P G S
                KKK_tag: . . . . . . . . . . H H H H G G G S L E V L F Q|G P G S K K K G S
                avi_tag: M A G L N D I F E A Q K I E W H E G S

N-terminal cap:
              Consensus: D L G K K L L E A A R A G Q D D E V R I L M A N G A D V N A
                 3G124: . Q . . . . . . . . . . . . . . . . . . . . . . . . . . . .
               3G124nc: . . . . . . . . . . . . . . . . . . . . . . . . . . . . . .
            nl3G124nc. . . . R M . . . . . . . . . . . . . . . . . . . . . . . . .
                  3G61: . . . . . . . . . . . . . . . . . . . . . . . . . . . . . .
                  YKKD: - - - - - - - - - - - - - - - - - - - - - - - - - - . . . .
                  YRLK: - - - - - - - - - - - - - - - - - - - - - - - - - - . . . .
                  YRID: - - - - - - - - - - - - - - - - - - - - - - - - - - . . . .
                nlYRLK: - - - - - - - - - - - - - - - - - - - - - - - - - - . . . .

First internal repeat:
              Consensus: X D X X G X T P L H L A A X X G H L E I V E V L L K Z G A D V N A
                 3G124: A . D V . V . . . . . . . Q R . . . . . . . . . . . C . . . . . .
               3G124nc: A . D V . V . . . . . . . Q R . . . . . . . . . . . Y . . . . . .
            nl3G124nc. A . D V . V . . . . . . . Q R . . . . . . . . . . R Y . . . . . .
                  3G61: L . E V . W . . . . . . . W - . . . . . . . . . . . N . . . . . .
                  YKKD: Y . E V . W . . . . K . . W - . . . . K . . D . . . N . . . . . .
                  YRLK: Y . E V . W . . . . R . . W - . . . . L . . K . . . N . . . . . .
                  YRID: Y . E V . W . . . . R . . W - . . . . I . . D . . . N . . . . . .
                nlYRLK: Y . E V . W . . . . R . . W - . . . . L . . R . . R N . . . . . .

Second internal repeat:
              Consensus: X D X X G X T P L H L A A X X G H L E I V E V L L K Z G A D V N A
                 3G124: A . L W . Q . . . . . . . T A . . . . . . . . . . . N . . . . . .
               3G124nc: A . L W . Q . . . . . . . T A . . . . . . . . . . . N . . . . . .
            nl3G124nc. A . L W . Q . . . . . . . T A . . . . . . . . . . R N . . . . . .
                  3G61: A . I D . Y . . . . . . . F S . . . . . . . . . . . Y . . . . . .
                  YKKD: A . I D . Y . . . . . . . F S . . . . . . . . . . . Y . . . . . .
                  YRLK: A . I D . Y . . . . . . . F S . . . . . . . . . . . Y . . . . . .
                  YRID: A . I D . Y . . . . . . . F S . . . . . . . . . . . Y . . . . . .
                nlYRLK: A . I D . Y . . . . . . . F S . . . . . . . . . . R Y . . . . . .

Third internal repeat:
              Consensus: X D X X G X T P L H L A A X X G H L E I V E V L L K Z G A D V N A
                 3G124: R . N I . H . . . . . . . W A . . . . . . . . . . . Y . . . . . .
               3G124nc: R . N I . H . . . . . . . W A . . . . . . . . . . . Y . . . . . .
            nl3G124nc. R . N I . H . . . . . . . W A . . . . . . . . . . R Y . . . . . .
                  3G61: D . Q A . F . . . . . . . I F . . . . . . . . . . . N . . . . . .
                  YKKD: D . Q A . F . . . . . . . I F . . . . . . . . . . . N . . . . . .
                  YRLK: D . Q A . F . . . . . . . I F . . . . . . . . . . . N . . . . . .
                  YRID: D . Q A . F . . . . . . . I F . . . . . . . . . . . N . . . . . .
                nlYRLK: D . Q A . F . . . . . . . I F . . . . . . . . . . R N . . . . . .

C-terminal cap:
             old C-cap: Q D K F G K T A F D I S I D N G N E D L A E I L Q - - -
      stabilized C-cap: . . . . . . . P . . L A . . . . . . . I . . V . . K A A
                 3G124: . . . . . . . . . . . . . . . . . . . . . . . . . - - -
               3G124nc: . . . . . . . P . . L A . . . . . . . I . . V . . K A A
            nl3G124nc. . . R . . H . P . . L A . . . . . . . I . . V . . R A A
                  3G61: . . . . . . . . . . . . . . . . . . . . . . . . . . . .
                  YKKD: . . . . . . . P . . L A . . . . . . . I . . V . . K A A
                  YRLK: . . . . . . . P . . L A . . . . . . . I . . V . . K A A
                  YRID: . . . . . . . P . . L A . . . . . . . I . . V . . K A A
                nlYRLK: . . R . . H . P . . L A . . . . . . . I . . V . . R A A

C-terminal tags:
His6 (when avi_tag is used): K L N H H H H H H
                         GGC: K L N G G C

Linker:
                   GS7: G G G S G G G
                  GS11: . . . . . . . S G G G
                   GC7: . . . C . . .
```

Nomenclature of GFP-clamps (e.g.):
gc_R7: 3G124nc-GS**7**-Y**R**LK
gc_R11: 3G124nc-GS**11**-Y**R**LK
gc_K11: 3G124nc-GS**11**-Y**K**KD
nl_gc_R7: **nl**3G124nc-GS**7**-**nl**Y**R**LK
nl: no lysine

Nomenclature with tags (e.g.):
gc_R7: MRGS_His10_3C_R7 (standard expression is with His10_tag which has been cleaved off by 3C-protease)
3×cys_gc_R7: MRGS_His6_GCG_R7_GGC with GC7-linker (3 cysteine introduced**)**

avi_gc_R7: **avi**_tag_R7_His6
KKK_nl_gc_R7: **KKK**_tag_nlR7 (3C-cleaved)

X: Randomized position to all amino acids except C and P
Z: Randomized position to only N, H or Y

SI Figure 1. Sequence alignment of designed GFP-binding DARPins. The top row indicates the consensus sequence with randomized positions indicated as X (randomization to all amino acids but Cys and Pro) and Z (randomization to Asn, His or Tyr) highlighted in

black frames. Identical residues are shown as dots (.), gaps are shown as hyphens (-). Differences to the consensus sequence are shown in one-letter amino acid code. Mutations introduced after truncation of the N-cap of 3G61 are highlighted in grey. Mutations introduced to replace lysines in gc_R7 are highlighted in blue. The recognition sequence of 3C-protease is shown in green with the cutting site as a vertical line.

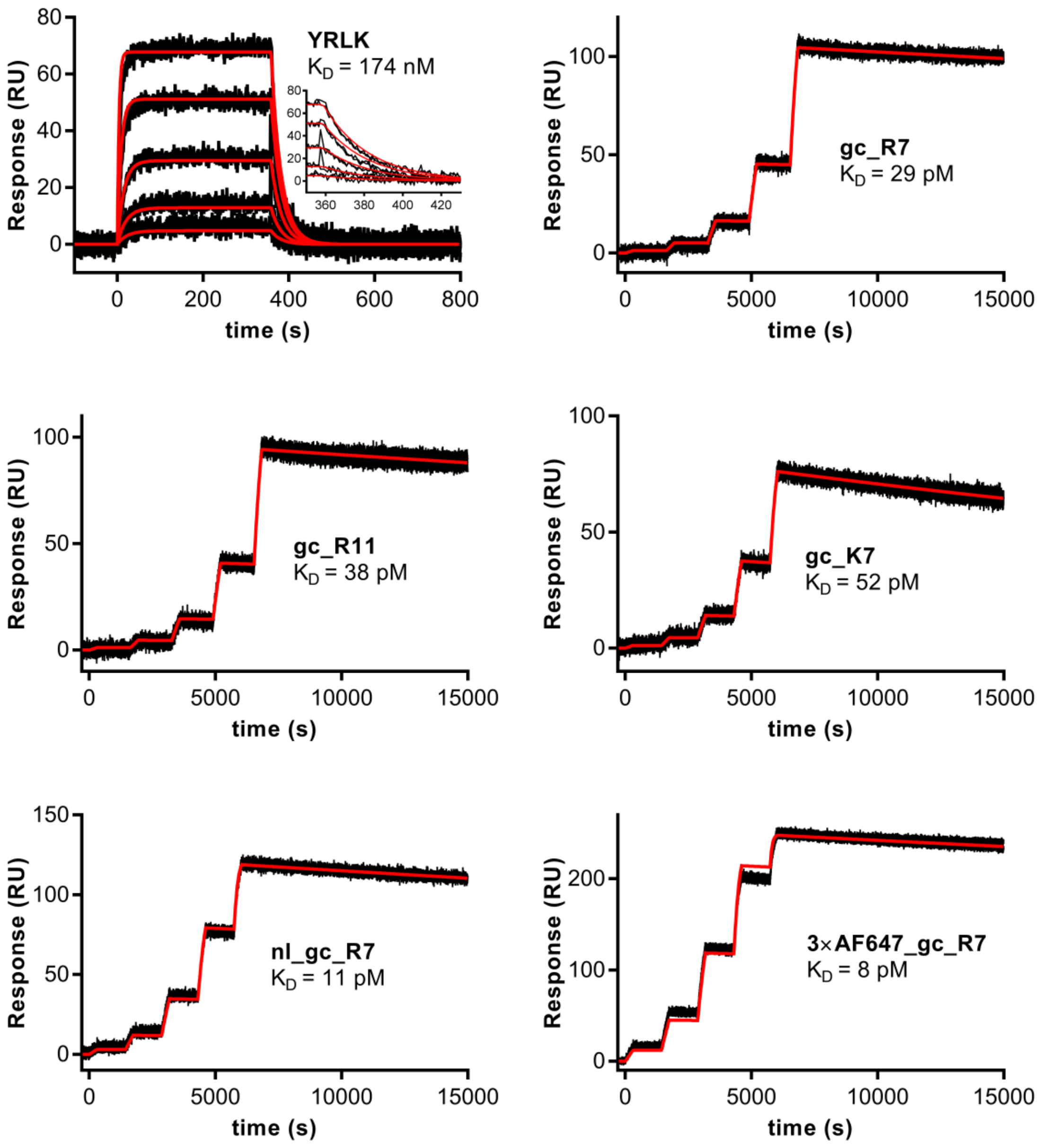

SI Figure 2. SPR measurements of GFP-binding DARPins and fusions. Biotinylated GFP was immobilized on a neutravidin sensor surface. YRLK was injected at concentrations of 11, 33, 100, 300 and 900 nM and fitted to a Langmuir binding model. 3×AF647_gc_R7 was injected at concentrations of 0.22, 0.66, 2, 6 and 18 nM and fitted to a kinetic titration model. All other constructs were injected at concentrations of 0.11, 0.33, 1, 3 and 9 nM and fitted to a kinetic titration model.

```
          1        10        20        30        40        50
    GFP: MVSKGEE----LFTGVVPILVELDGDVNGHKFSVSGEGEGDATYGKLTLKF  47
  sfGFP: .......----.......................R........N.......  47
   eGFP: .......----........................................  47
   eYFP: .......----........................................  47
   eCFP: .......----........................................  47
mCherry: .......DNMAIIKEFMRFK.HME.S....E.EIE.....RPYE.TQ.A.L  51
 mRuby2: .......----.IKENMRMK.VME.S....Q.KCT.....NPYM.TQ.MRI  47

         51        60        70        80        90        100
    GFP: ICTTG-KLPVPWPTLVTTFTYGVQCFSRYPDHMKRHDFFKSAMPEGYVQE  96
  sfGFP: .....-............L...............................  96
   eGFP: .....-............L...............Q...............  96
   eYFP: .....-.............G..L...A.......Q...............  96
   eCFP: .....-............L.W.V...S.......Q...............  96
mCherry: KV.K.GP..FA.DI.SPQ.M..SKAYVKH.ADIP--.YL.LSF...FKW.  99
 mRuby2: KVIE.GP..FAFDI.ATS.M..SRT.IK..KGIP--.FF.QSF...FTW.  95

         101       110       120       130       140       150
    GFP: RTIFFKDDGNYKTRAEVKFEGDTLVNRIELKGIDFKEDGNILGHKLE-YN  145
  sfGFP: ...S.....T.....................................-..  145
   eGFP: ...............................................-..  145
   eYFP: ...............................................-..  145
   eCFP: ...............................................-..  145
mCherry: .VMN.E.G.VVTVTQDSSLQDGEFIYKVK.R.TN.PS..PVMQK.TMGWE  149
 mRuby2: .VTRYE.G.VVTVMQDTSL.DGC..YHVQVR.VN.PSN.PVMQK.TKGWE  145

         151       160       170       180       190       200
    GFP: YNSHNVYIMADKQKNGIKVNFKIRHNIEDG---SVQLADHYQQNTPIGDG  192
  sfGFP: F.......T.........A.......V...---.................  192
   eGFP: ..............................---.................  192
   eYFP: ..............................---.................  192
   eCFP: .I......T.........A...........---.................  192
mCherry: AS.ERM.----PEDGALKGEIKQ.LKLK..GHYDAEVKTT.KAKK.V---  192
 mRuby2: PNTEMM.----PADG.LRGYTHMALKVDG.GHL.CSFVTT.RSKKTVGN-  190

         201       210       220       230       240       250
    GFP: PVLLPDNHYLSTQSALSKDPNEKRDHMVLLEFVTA----AGITHGMDELYK  239
  sfGFP: ..............V....................----............  239
   eGFP: ...................................----....L.......  239
   eYFP: ...........Y.......................----....L.......  239
   eCFP: ...................................----....L.......  239
mCherry: --Q..GAYNVNIKLDITS---HNEDYTIVEQYER.EGRHST--G.......  236
 mRuby2: -IKMPGI.AVDHRLERLE---.SDNE.FVVQREH.VAKFA.LGG.......  237
```

SI Figure 3. Sequence alignment of fluorescent proteins used in this study. The top row indicates the sequence of GFP including the chromophore mutation S65T. Identical residues are shown as dots (.), gaps are shown as hyphens (-). Differences to the consensus sequence is shown in one-letter amino acid code and highlighted in light green (sfGFP), dark green (eGFP), yellow (eYFP) and cyan (eCFP). For mCherry and mRuby2, residues that differ from each other are highlighted in grey.

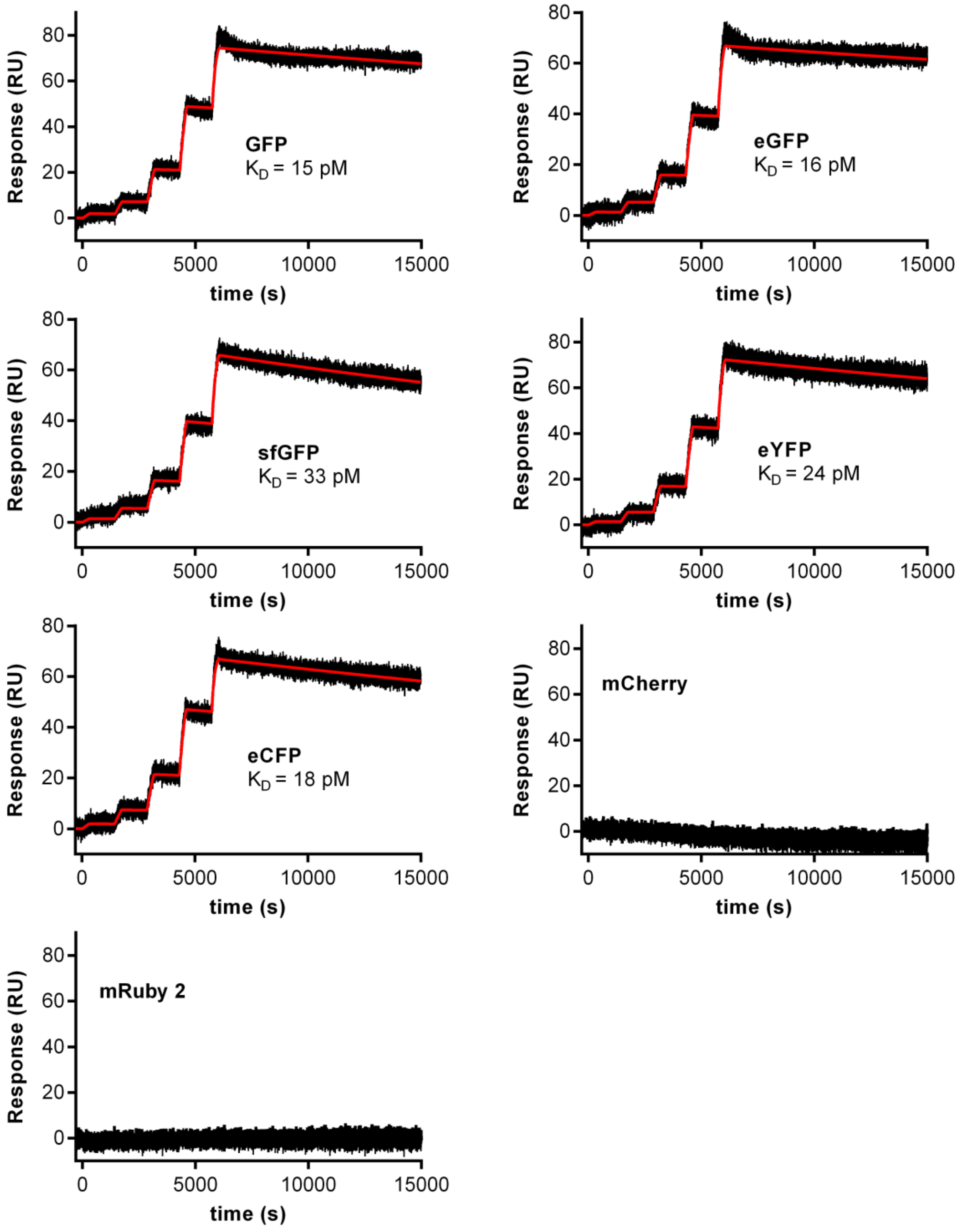

SI Figure 4. SPR measurements of different FPs to gc_R7. Biotinylated gc_R7 was immobilized on a Neutravidin sensor chip. All FPs were injected at concentrations of 0.11, 0.33, 1, 3 and 9 nM and fitted to a kinetic titration model (no fit for mCherry and mRuby2).

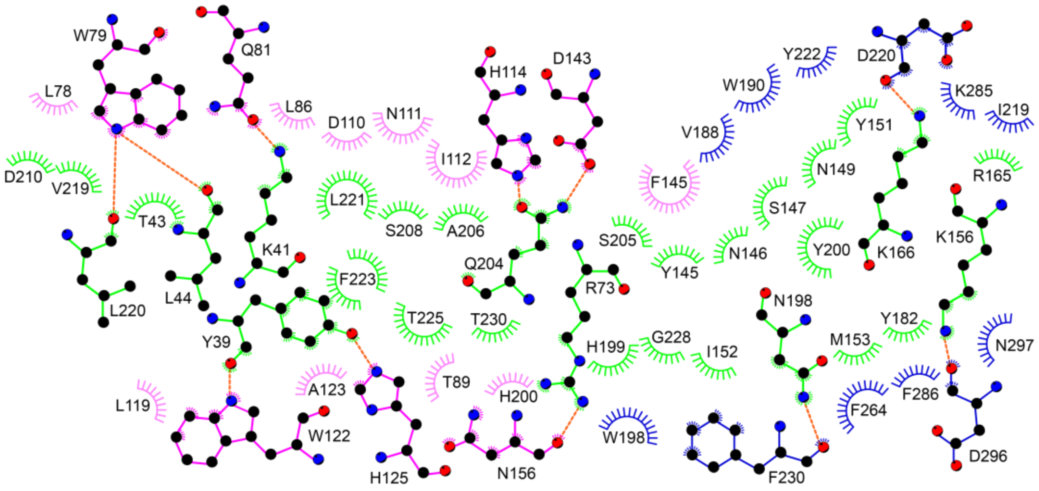

SI Figure 5. Detailed interaction map between gc_K11 and eGFP (PDB ID: 5MA5, chains B and C). eGFP residues are shown in green, residues of the 3G124nc domain in pink and of the YKKD domain in blue, hydrogen bonds are shown in orange (prepared with LigPlot+).

SI Tables

SI Table 1: Crystallographic data collection and refinement statistics

Complex<br>PDB-ID	3G124nc:eGFP<br>5MA6	3G124nc:eGFP<br>5MA8	3G61:eGFP<br>5MAD
Crystalization condition	0.5 M KH_2PO_4<br>0.1 M Na-acetate pH 5.5	25% PEG 2K MME<br>0.3 M Na-acetate<br>0.1 M Tris (HOAc) pH 7.5	30% PEG 4000<br>0.2 M Ammonium acetate<br>0.1 M tri-Na-citrate pH 5.5
Data collection			
Resolution range (Å)	50.01 - 2.30	43.94 - 2.35	48.76 - 1.53
Space group	$P6_122$	$P4_1$	$P2_1$
Molecules/AU	2 (1 complex)	4 (2 complexes)	8 (4 complexes)
Unit cell parameters			
a, b, c (Å)	70.31, 70.31, 432.72	62.14, 62.14, 213.20	60.42, 83.07, 162.00
α, β, γ (°)	90, 90, 120	90, 90, 90	90, 94.59, 90
Unique reflections	29482	33548	237243
Multiplicity	37.3 (40.7)	13.9 (14.1)	6.7 (6.8)
Completeness	98.7 (98.5)	99.9 (100.0)	98.8 (99.5)
R_{merge}	0.148 (8.52)	0.107 (1.28)	0.047 (1.69)
$<I>/\sigma(I)$	17.55 (0.68)	22.33 (2.78)	15.79 (1.39)
CC(1/2)	1.00 (0.42)	0.99 (0.81)	0.99 (0.75)
Wilson B-factor ($Å^2$)	70.34	42.49	24.37
Refinement			
R_{work}(%)	0.205	0.189	17.1
R_{free}(%)	0.241	0.236	19.9
RMSD of bond lengths	0.024	0.004	0.006
RMSD of bond angles	2.559	0.890	0.838
Average B-factor ($Å^2$)	84.69	55.36	39.5
Ramachandran plot (%)			
favored	94.99	99.07	98.16
allowed	5.01	0.93	1.71
outliers	0.00	0.00	0.13
Non-hydrogen atoms			
protein	2980	5922	12286
ligands	51	44	145
waters	45	181	1246

Statistics for highest resolution shell in parentheses

SI Table 1 (continued): Crystallographic data collection and refinement statistics

Complex	gc_K7:eGFP	gc_K11:eGFP	gc_R7:eGFP
PDB-ID	5MA4	5MA5	5MAK
Crystalization condition	30% w/v PEG 4000<br>0.2 Na-acetate<br>0. 1M Tris (HCl) pH 8.5	20% w/v PEG 4000<br>20% v/v 2-Propanol<br>0.1 M tri-Na-citrate pH 5.6	20% w/v PEG 4000<br>20% v/v 2-Propanol<br>0.1 M tri-Na-citrate pH 5.6
Data collection			
Resolution range (Å)	47.29 - 1.40	44.25 - 1.85	43.53 - 2.50
Space group	$P2_1$	P1	P1
Molecules/AU	2 (1 complex)	4 (2 complexes)	4 (2 complexes)
Unit cell parameters			
a, b, c (Å)	55.88, 92.34, 56.53	58.71, 60.28, 90.24	57.96, 61.38, 89.38
α, β, γ (°)	90, 114.6, 90	86.94, 79.13, 89.35	93.12, 102.74, 94.76
Unique reflections	102263	92746	39327
Multiplicity	5.4 (5.2)	3.7 (3.8)	3.5 (3.7)
Completeness	99.7 (99.7)	89.5 (89.2)	95.1 (97.2)
R_{merge}	0.053 (0.89)	0.044 (0.65)	0.141 (0.86)
$<I>/\sigma(I)$	17.60 (2.1)	18.31 (2.46)	8.62 (1.86)
CC(1/2)	0.99 (0.74)	0.99 (0.81)	0.99 (0.78)
Wilson B-factor ($Å^2$)	15.5	27.04	36.38
Refinement			
R_{work}(%)	14.2	15.4	25.7
R_{free}(%)	17.0	18.4	30.4
RMSD of bond lengths	0.014	0.007	0.002
RMSD of bond angles	1.275	0.859	0.506
Average B-factor ($Å^2$)	25.8	41.0	57.1
Ramachandran plot (%)			
Favored	98.07	98.54	95.94
allowed	1.93	1.37	3.87
outliers	0.00	0.1	0.2
Non-hydrogen atoms			
protein	4363	8044	7922
ligands	29	118	109
waters	725	779	143

Statistics for highest resolution shell in parentheses

SI Table 1 (continued): Crystallographic data collection and refinement statistics

Complex	gc_R11:eGFP	gc_R11:eGFP
PDB-ID	5MA3	5MA9
Crystalization condition	30% w/v PEG 8000	30% w/v PEG 4000
	0.2 M Na-acetate	0.2 M Li_2SO_4
	0.1 M Na-cacodylate pH 6.5	0.1 M Tris pH 8.5
Data collection		
Resolution range (Å)	48.98 - 1.70	44.68 – 1.57
Space group	$P2_1$	P1
Molecules/AU	2 (1 complex)	8 (4 complexes)
Unit cell parameters		
a, b, c (Å)	59.85, 90.61, 60.92	81.88, 89.89, 90.04
α, β, γ (°)	90, 103.4, 90	95.84, 116.53, 92.35
Unique reflections	67771	302440
Multiplicity	6.7 (6.9)	3.4 (3.6)
Completeness	97.6 (98.6)	95.2 (94.4)
R_{merge}	0.105 (3.44)	0.046 (0.57)
$\langle I\rangle/\sigma(I)$	10.47 (0.98)	14.03 (2.6)
CC(1/2)	0.99 (0.56)	0.99 (0.75)
Wilson B-factor (Å^2)	27.6	18.1
Refinement		
R_{work}(%)	16.1	15.8
R_{free}(%)	19.2	20.3
RMSD of bond lengths	0.007	0.005
RMSD of bond angles	0.851	0.929
Average B-factor (Å^2)	38.9	32.0
Ramachandran plot (%)		
favored	98.09	98.54
allowed	1.91	1.36
outliers	0.00	0.10
Non-hydrogen atoms		
protein	4053	16022
ligands	46	132
waters	393	2095

Statistics for highest resolution shell in parentheses
